# Single photon emission computed tomography/computed tomography imaging of gouty arthritis: A new voice

**DOI:** 10.2478/jtim-2022-0066

**Published:** 2023-03-19

**Authors:** Yan Wang, Yan Zha, Lin Liu, Ang Liao, Ziqiang Dong, Neil Roberts, Yaying Li

**Affiliations:** Department of Nuclear Medicine, Guizhou Provincial People's Hospital, Affiliated Hospital of Guizhou University, Guiyang 550002, Guizhou Province, China; Departnent of Nephrology, Guizhou Provincial People's Hospital, Affiliated Hospital of Guizhou University, Guiyang 550002, Guizhou Province, China; Department of Respiratory and Critical Care Medicine, Guizhou Provincial People's Hospital, Affiliated Hospital of Guizhou University, Guiyang 550002, Guizhou Province, China; School of Clinical Sciences, The Queen’s Medical Research Institute, University of Edinburgh, Edinburgh EH8 9YL , United Kingdom

**Keywords:** gout, ^99m^Tc, single photon emission computed tomography/computed tomograph, chronic kidney disease, radiopharmaceutical, advanced

## Abstract

Gouty arthritis, often referred to simply as gout, is a disorder of purine metabolism characterized by the deposition of monosodium urate monohydrate (MSU) crystals in multiple systems and organs, especially in joints and their surrounding soft tissue. Gout is a treatable chronic disease, and the main strategy for effective management is to reverse the deposition of MSU crystals by uric acid reduction, and to prevent gout attacks, tophi deposition and complications, and thereby improve the quality of life. However, the frequent association of gout with other conditions such as hypertension, obesity, cardiovascular disease, diabetes, dyslipidemia, chronic kidney disease (CKD) and kidney stones can complicate the treatment of gout and lead to premature death. Here, we review the use of medical imaging techniques for studying gouty arthritis with special interest in the potential role of single photon emission computed tomography (SPECT)/computed tomography (CT) in the clinical management of gout and complications (*e.g*., chronic kidney disease and cardiovascular disease).

## Introduction

Gout, characterized by the deposition of monosodium urate monohydrate (MSU) crystals in synovial fluid, joint spaces and other soft tissues, is a disorder of purine metabolism, caused by prolonged hyper-uricemia secondary either to overproduction, or renal and intestinal under-excretion of uric acid (UA).^[1,2]^ Gout can occur in multiple systems and organs, especially in joints and their surrounding soft tissue. The most commonly affected sites are the meta-tarso-phalangeal (MTP) joint, followed by the elbow, knee, and inter-knuckle joints. Previously, it was thought that the axial skeleton was only very occasionally involved. However, based on the findings reported in recent publications, the incidence of spinal gout may be more common than that was first thought.^[3, 4, 5]^ In addition, gout may be associated with the development of other conditions such as hypertension, obesity, cardiovascular disease, diabetes, dyslipidemia, chronic kidney disease (CKD) and kidney stones,^[6, 7, 8, 9, 10, 11, 12]^ which can complicate the treatment of gout and lead to premature death.^[13]^

Here, we review the use of medical imaging techniques for studying gouty arthritis with special interest in the potential role of single photon emission computed tomography (SPECT)/computed tomography (CT). SPECT/CT is a molecular imaging technology that is widely used in clinical research offering the main advantage that information regarding functional metabolism is integrated with information regarding anatomical structure. The advance of digital image analysis software for use with SPECT/CT offers particular advantages for studying gouty arthritis.

## Epidemiology

Gout is the most common inflammatory arthritis occurring in adults.^[14]^ Information from population-based studies carried out of adults in Asia, Europe and North America indicates that the incidence of gouty arthritis is between 0.6 and 2.9 per 1000 person-years, and appears to be higher in western countries than in Asia, which is likely related to lifestyle diets (*e.g*., obesity, alcohol consumption), environmental factors and genetics. The corresponding prevalence is between 0.68% and 3.90%,^[7,9,15, 16, 17, 18]^ being highest at 6.1% in Oceania (Maori ancestry),^[1]^ followed by 3.9% in USA, 3.2 % in New Zealand (European ancestry), 1.4%–2.5% in the UK, 1.4% in Germany, 1%–3% in China,^[19]^ 1.94% in Korea^[14]^ and 0.9% in France.

Gout is more common in men than in women, with the male to female sex ratio reported to range between 2:1 and 4:1 in Europe and North America and being as high as 8:1 in Asia.^[20]^ Interestingly, it has been reported that serum urate concentrations are lower in premenopausal women due to the uricosuric effects of estrogen, and following the menopause, urate increases to concentrations similar to those observed in men.^[21]^

## Pathophysiology

Clinically, gout is characterized by painful flares of acute monoarthritis interspersed with asymptomatic periods, and the course of the disease has been classified into four stages, namely: (1) asymptomatic hyper-uricemia, (2) acute gouty attack, (3) inter-critical period and (4) chronic tophaceous gout.^[20]^ High serum uric acid is the most important risk factor for the development of gout. Exposure of extremities to low temperatures, physiological pH between 7 and 10, high concentration of sodium ions, and synovial and cartilage components can all promote monosodium urate crystallization,^[22,23]^ especially in the first MTP joint. The biomechanical load on the foot and ankle during the normal gait cycle produces a unique pattern of crystal deposition with clustering at pressure points within the joint.^[24]^ Within the joints, reaction between monosodium urate crystals and the nucleotide-binding oligomerization domain, leucine-rich repeat and pyrin domain-containing 3 inflammasome (NLRP3) in macrophages and monocytes leads to a series of inflammatory responses. The resulting inflammatory products^[20,25]^ (*e.g*., neutrophile granulocyte, interleukin [IL]-10, IL-1ra, transforming growth factor [TGF]-β and IL-37) and persistent hyper-uric acid produce chronic inflammation leading to formation of tophi.^[20]^ The common feature of advanced gout is structural bone and joint damage (bone erosion and focal cartilage damage) caused by tophi.

Oxidative stress represents a fundamental pathway in diseases related to hyper-uricemia (hypertension and diabetes), and in the development of damage to the heart and cardiovascular system.^[8]^ Gout can also affect the kidneys with UA being deposited as crystals in the renal tubules causing an increase in oxidative stress, tubule-interstitial inflammation with afferent arteriopathy of the arteriole and hyperplasia/hypertrophy of the tunica muscularis,^[26]^ activation of the renin-angiotensin-aldosterone system^[27]^ and impairment in endothelial function due to a reduction of nitric oxide levels.^[28]^ It is well known that renal damage is a risk factor for CV. However, one of the most important mechanisms through which UA is probably related to CV events is renal damage. Regarding metabolic derangement, UA is involved in the deamination of adenosine monophosphate, resulting in increased fat accumulation, which is one of the steps at the basis of hyper-insulinemia, and consequently in insulin resistance.^[29]^

## Imaging

**Figure 1:**
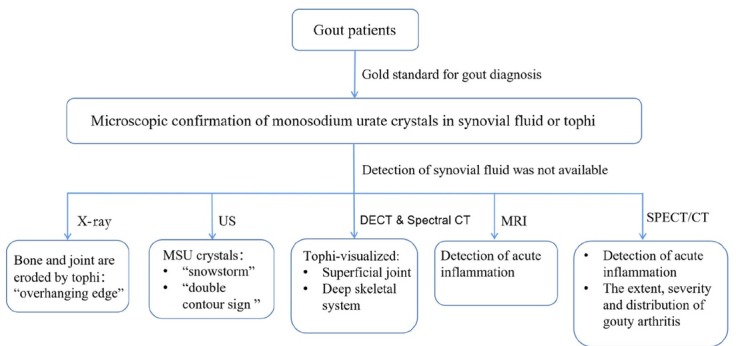
A flowchart of demonstration for imaging of gout. MRI: magnetic resonance imaging; MSU: monosodium urate monohydrate; DECT: dual energy computed tomography; US: ultrasound; SPECT: single photon emission computed tomography; CT: computed tomography.

The diagnosis of gout is usually based on clinical presentation, laboratory tests and imaging. With regard to the latter, in 2015, the American College of Rheumatology and European League Against Rheumatism^[1]^ proposed that microscopic confirmation of monosodium urate crystals in synovial fluid or tophi be the gold standard for gout diagnosis. Imaging techniques are also recommended for evaluation of gout,^[1,30]^ and ultrasound (US) imaging and dual energy computed tomography (DECT) are often used. We have prepared a flowchart of demonstration for imaging of gout (Figure 1), and a summary of research on advanced imaging of gout in 2012–2021 can be found in Table 1.

**Table 1:** The research on advanced imaging of gout in 2012–2021

Title	Journal	Study nature	Study sample size	Study time	Study result
Tips and tricks to recognize microcrystalline arthritis^[31]^	*Rheumatology*	Review	—	2012	US detects not only urate crystals but also tophaceous aggregates both around and inside the erosions, which is considered the most operator-dependent imaging technique.
Ultrasound-detected musculoskeletal urate crystal deposition: which joints and what findings should be assessed for diagnosing gout?^[32]^	*Annals Of The Rheumatic Diseases*	Prospectively research	133	2013	US bilateral assessment of one joint, three articular cartilages and two tendons may be valid for diagnosing gout with acceptable sensitivity and specificity.
Artifacts in Dual-Energy CT Gout Protocol: A Review of 50 Suspected Cases With an Artifact Identification Guide^[33]^	*American Journal Of Roentgenology*	Prospective study	50	2013	Artifacts, although common in DECT gout protocol, can usually be readily recognized, thereby avoiding false-positive results.
Imaging Modalities for the Classification of Gout: Systematic Literature Review and Meta-Analysis^[34]^	*Annals Of The Rheumatic Diseases*	Systematic Literature Review and Meta-Analysis	Eleven studies (9 manuscripts and 2 meeting abstracts) satisfied the inclusion criteria	2015	US and DECT show promise for gout classification but the few studies to date have mostly been in patients with longstanding, established disease. The contribution of imaging over clinical features for gout classification criteria requires further examination.
2015 Gout classification criteria: an American College of Rheumatology/ European League Against Rheumatism collaborative initiative^[1]^	*Annals Of The Rheumatic Diseases*	Criteria	—	2015	US and DECT are recommended as one of the gout scoring criteria.
Performance of Ultrasound in the Diagnosis of Gout in a Multi-Center Study: Comparison with Monosodium Urate Crystal Analysis as the Gold Standard^[35]^	*Arthritis & Rheumatology*	Multi-Center Study	824	2016	US features of MSU crystal deposition had high specificity and high positive predictive value but more limited sensitivity for early gout. The specificity remained high in subjects with early disease and without clinical signs of tophi.
The performance of dual-energy CT in the classification criteria of gout: a prospective study in subjects with unclassified arthritis^[36]^	*Rheumatology*	Original article	89	2019	DECT seems to have an additive value in gout classification, especially when microscopy of synovial fluid is negative.
Ultrasound for the diagnosis of gout—the value of gout lesions as defined by the Outcome Measures in Rheumatology ultrasound group^[37]^	*Rheumatology*	Original article	82	2020	US-visualized tophus show high specificities, positive predictive values and accuracies for diagnosing gout, and are therefore valid tools in clinical practice.
Dual-energy computed tomography versus ultrasound, alone or combined, for the diagnosis of gout: a prospective study of accuracy^[38]^	*Rheumatology*	prospective study	147	2021	Feet/ankles or knees DECT alone had the best overall accuracy for gout diagnosis. DECT/US combination or multiple joint imaging offered no additional increase in overall diagnostic accuracy.

MSU: monosodium urate monohydrate; DECT: dual energy computed tomography; US: Ultrasound; SPECT: single photon emission computed tomography.

### X-ray and X-ray CT

Generally speaking, many of the abnormalities reported on plain film X-rays of the spine obtained for patients with gout are non-specific such as cervical spondylosis,^[39]^ spondylolisthesis^[40]^ and degenerative change.^[41]^ However, if the bone and joint are eroded by tophi, there will be typical punched out erosion. Another characteristic feature is the presence of a so-called “overhanging edge”. This is a thin bony extension at the periphery of the erosion protruding into the soft tissues and partially covering the tophus.^[42]^ Images obtained by using X-ray CT are more useful for evaluating the spine and sacroiliac joints than X-ray, and can also be used to measure tophi density, which typically has a mean density of 160–170 Hounsfield units.^[42,43]^ However, as will be described below, ultrasound and DECT imaging,^[1,20]^ rather than X-ray or X-ray CT, are the main imaging techniques for evaluating gout.

### US

Several features of early gout may be seen on ultrasound images,^[1,44,45]^ including effusion in the affected joint and synovial hypertrophy and bursitis, which are often seen but are non-specific; MSU crystals of less than 1 mm, and larger MSU aggregates, are referred to as micro-tophi, which have what is described as a “snowstorm” appearance. However, the most characteristic appearance of gout on ultrasound images is the “double contour sign (DCS)”, which results from deposition of gout crystals on the surface of the articular cartilage. The crystals eventually create a continuous hyper-echoic line overlying the articular cartilage, paralleling the subchondral bone. Recently, several longitudinal studies have demonstrated good feasibility of ultrasound as a tool for measuring changes in tophi after gout treatment.^[46,47]^ The use of ultrasound in the assessment of rheumatologic conditions is increasing due to increasing availability, relative low cost, lack of ionizing radiation, dynamic and multi-planar imaging capability, and high soft tissue resolution,^[48]^ but ultrasound also has limitations. Intraosseous tophi cannot be detected using ultrasound due to the inability of ultrasound to penetrate bone cortex.^[45]^ Therefore, US has a limited role in studying deep lying regions of the musculoskeletal system (*i.e*., axial skeleton).^[45]^

### DECT and Spectral CT

When combined with image processing software which includes a material decomposition algorithm to color-code urate, DECT dual energy imaging provides a sensitive method for the detection of tophi.^[49,50]^ Different manufacturers have developed slightly different system and algorithms, *i.e*., dual-source CT with 80 (100) kVp and 140 kVp tubes (Siemens Medical Solution),^[49]^ dual-layer multi-detector scanner with acquisition 120 or 140 kVp (Philips Healthcare)^[51]^ and CT unit with one rapid kVp switching source and new detector based on gemstone scintillator materials (GE Healthcare)^[50]^ and particular range of colors to depict urate crystals at articular or periarticular sites. MSU crystals of < 2 mm can be detected earlier by DECT than by using other imaging methods, and additionally, DECT allows bone erosion to be well depicted.^[46]^ Compared with ultrasound, DECT is more suitable for the detection of gout spondylitis. However, DECT does not perform well when inflammation is present.^[52]^ In addition, artifacts may arise from nail beds, the so-called clumpy artifacts along the tendon, calcification, movement and beam hardening, leading to false-positive results and volumetric analysis appears not to show change over time, even when therapy is effective.^[53,54]^ Furthermore, the required image post-processing is a time-consuming and potentially costly process. DECT, actually, is a robust tool to detect tophi, which has been used to measure size of tophi and to evaluate the dissolution of local joint MSU crystals after treatment. Currently, however, guidelines do not exist with regard to the measurement technique to be adopted,^[45]^ and there may be a possibility of large deviations due to subjective factors, especially in the evaluation of treatment response.

### Magnetic resonance imaging

Magnetic resonance imaging (MRI) is the preferred imaging modality for evaluating the spinal canal. Gout in the spinal canal is extremely rare and difficult to diagnose because its clinical manifestation and radiologic findings mimic tumors, abscess, tuberculosis and degenerative spinal diseases.^[55,56]^ Generally speaking, differential diagnosis can be made by clinical symptoms. In particular, spinal tumors may be associated with severe local pain, spinal abscesses may be associated with a history of fever and spinal tuberculosis may have a history of tuberculosis infection of other organs. Elgafy *et al*.^[57]^ performed a systematic review and reported that the majority of spinal tophi were hypo-intense on both T1-and T2-weighted MR images (45.5% and 26.5%, respectively) and 47.1% showed gadolinium enhancement which occurs as a result of vascularized reactive granulation. Although the MR appearance of tophi is non-specific, the diagnosis of gout should be considered when a mass has heterogeneous low to intermediate signal intensity on T2-weighted images, especially if the mass erodes adjacent bones.^[42]^

### SPECT/CT

**Figure 2:**
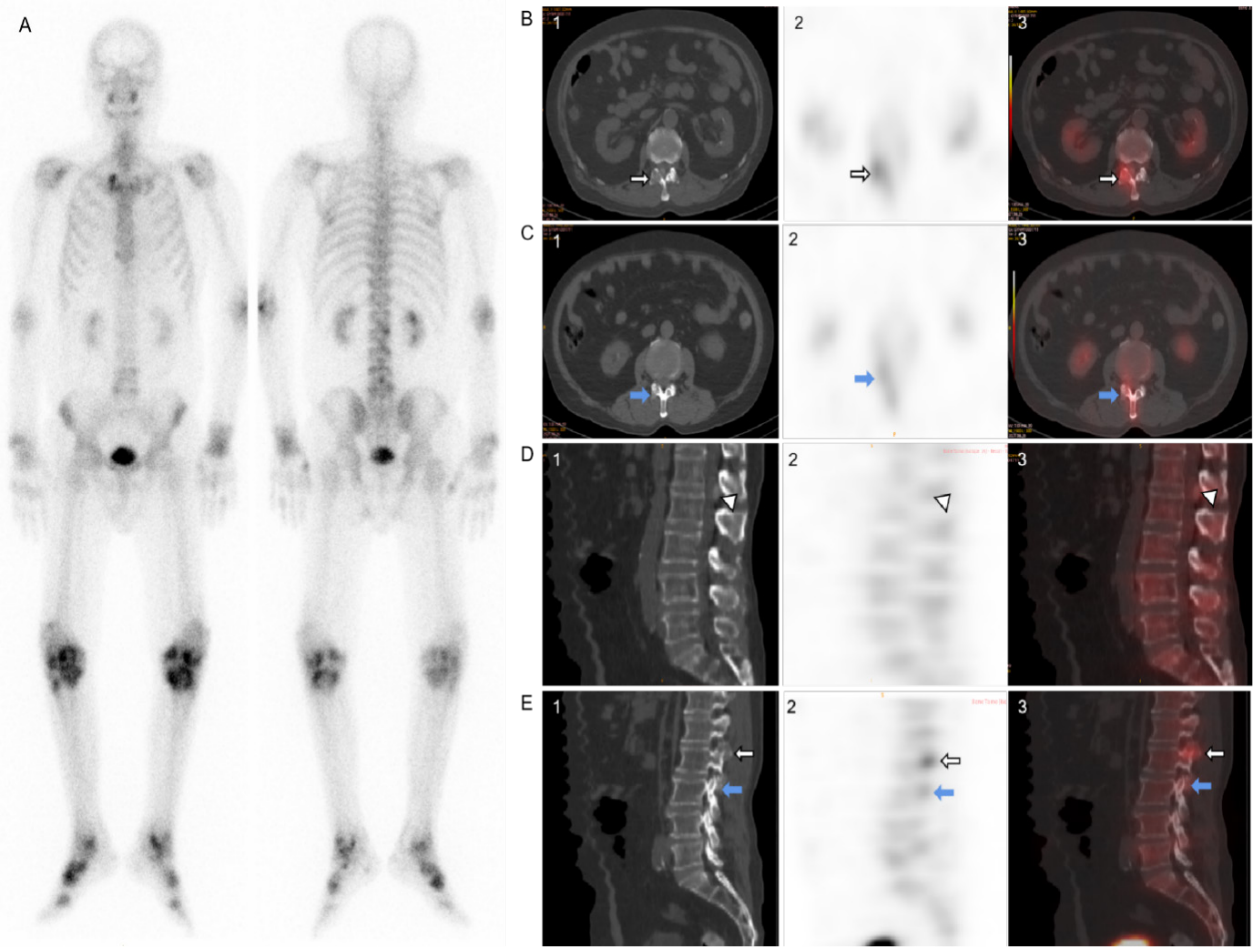
A 66-year-old elderly male. Postoperative pathological diagnosis of the lumbar spine: gout nodules. Anterior and posterior whole body views of ^99m^Tc-MDP skeletal scintigraphy are shown. (A) Multiple regions of anomalous uptake of ^99m^Tc-MDP were detected in the lumbar 1/2 right, right rotoclavicular, knee and ankle joints. (B–E) SPECT/CT can synchronize the case of bone, which enables the acquisition of synchronous imaging information of metabolism (focal anomalous uptake of ^99m^Tc-MDP can be seen in an intra-spinal canal nodule at level L1/2 [white triangle]) and bone destruction in the gouty lesion area (bone erosions can be seen in the right inter-apophyseal joints at levels L1/2 [white arrow] and L2/3 [blue arrow]).^[64]^ MDP: methylene diphosphonate; SPECT: single photon emission computed tomography; CT: computed tomography.

Traditionally, in nuclear medicine, bone imaging is performed using whole-body skeletal scintigraphy with ^99m^Tc phosphate compounds, which may be helpful in revealing the extent, severity and distribution of gouty arthritis.^[2,58]^ However, SPECT, and especially SPECT/ CT, provides increased sensitivity and specificity.^[59]^ The outstanding feature of these techniques is that they reveal not only the morphology of the bone but also functional information regarding blood supply and metabolism, changes in which may often precede morphological changes. ^99m^Tc-methylene diphosphonate (MDP) is a commonly used nuclear medicine tracer for bone imaging, and hydroxyapatite crystals can be detected by large amounts of ^99m^Tc-MDP being absorbed on the crystal surface. Tophi may also be detected *via* the appearance of bone erosion and bone remodeling on the CT image. Nevertheless, there are only a few reports of ^99m^Tc-MDP bone imaging having been used to diagnose gouty arthritis.^[60, 61, 62, 63]^ In one study, an anomalous increase of ^99m^Tc-MDP was reported in tophi and interpreted to be due to inflammation causing an increase in blood supply to the region (Figure 2).^[64]^ However, the increase of ^99m^Tc-MDP uptake is non-specific because it can also occur at sites of infection and in tumor, which has led to reluctance to use ^99m^Tc-MDP SPECT/ CT in studies evaluating gouty arthritis. Accordingly, advanced molecular imaging using ^99m^Tc-MDP SPECT/CT was not included in the criteria for classifying gouty arthritis developed jointly by the American Society of Rheumatology and the European Federation against Rheumatism in 2015 (Table 2).^[1,2]^

**Table 2:** 2015 ACR/EULAR gout classification criteria[1]

**Imaging**	**Categories**	**Score**
Imaging evidence of urate deposition in symptomatic (ever) joint or bursa: ultrasound evidence of double-contour sign^¶^ or DECT demonstrating urate deposition**	Present (either modality)	4
Imaging evidence of gout-related joint damage: conventional radiography of the hands and/or feet demonstrates at least 1 erosion^††^	Present	4

^¶^If imaging is not available, score these items as 0. **Presence of color-coded urate at articular or periarticular sites. Images should be acquired using a DECT scanner, with data acquired at 80 kV and 140 kV and analyzed using gout-specific software with a 2-material decomposition algorithm that color-codes urate. A positive scan is defined as the presence of color-coded urate at articular or periarticular sites. Nailbed, submillimeter, skin, motion, beam hardening and vascular artifacts should not be interpreted as DECT evidence of urate deposition. ^††^Erosion is defined as a cortical break with sclerotic margin and overhanging edge, excluding distal interphalangeal joints and gull-wing appearance. DECT: dual energy computed tomography; MSU: monosodium urate monohydrate; ACR: AmericanCollegeofRheumatology; EULAR: European League Against Rheumatism.

Recently, the potential of using SPECT/CT in the imaging assessment of other post-operative skeletal pain,^[65, 66, 67]^ such as involving the spine, hip, hand and foot, has been demonstrated. For example, SPECT/ CT has been shown to be highly effective and cost-saving in supporting the management of patients with painful total-knee arthroplasty.^[68,69]^ Further progress will require the development of novel radiopharmaceuticals offering improved measurement of pharmacokinetics (*e.g*., next-generation radio-bisphosphonates) or visualization of infection (*e.g*., ^99m^Tc-UBI-29-4).^[70]^ Importantly, SPECT/ CT provides not only functional metabolic imaging but also allows a detailed assessment of changes in bone morphology and pathophysiology during the evolution of lesions.

In the past, analysis of SPECT/CT images was primarily based on visual inspection, which was too subjective and lacked the objectivity necessary for routine use in clinical practice. The development of the PET/ CT technique provided inherent advantages for quantification with higher sensitivity and increased spatial resolution, and which has to some extent held back the development of SPECT/ CT. However, subsequent developments have meant that SPECT/CT is now recognized as an effective, versatile and mature imaging tool. In particular, advances in technology mean that SPECT/ CT systems are equipped with high-end multi-slice CT. In addition, introduction of iterative reconstruction techniques for CT has meant that doses of ionizing radiation have been reduced by up to 80% without loss of image quality. Furthermore, new multimodal reconstruction techniques have led to improvements in image resolution and the possibility of visualizing small changes in the structure of the skeleton.^[71]^ Furthermore, the virtual monochromatic reconstructions of high-energy photons (140 keV) on dual-energy SPECT/ CT systems mean that there is also less susceptibility to metal artifacts.^[72]^

The combination of appropriate radionuclide imaging agents and dedicated image processing software means that not only can MSU crystals be visualized but also gout metabolism can be measured. In 2017, the first SPECT/ CT system with a 360° ring-shaped gantry was introduced, equipped with 12 CZT-based elongated detectors that can be controlled so as to be positioned as close as possible to the patient.^[73]^ Although the findings are preliminary, there are indications that the new system provides significantly enhanced image resolution and contrast.^[70]^

High-end SPECT/CT devices combine robust techniques for correcting for photon scattering, photon attenuation and partial volume effect with enhanced image reconstruction algorithms (*e.g*., iterative reconstruction, metal artifact reduction algorithms), which represents an important milestone in the development of nuclear medicine. The accuracy of ^99m^Tc imaging is reported to be within 65% of the true radionuclide concentration.^[74,75]^ This brings the potential for quantitative assays to be performed in routine clinical practice.^[74,76]^ For example, measurement of the standardized uptake value (SUV) of bone lesions with SPECT/CT can allow longitudinal assessment of bone pathologies, allow assessment of the degree of abnormality in temporomandibular joint disease,^[77]^ drug-related necrosis of the jaw and knee osteoarthritis,^[78]^ distinguish between bone metastases and degenerative changes,^[79,80]^ and help predict prognosis for radionuclide therapy in patients with prostate cancer.^[81]^

Worldwide, approximately 85% of all nuclear medicine screening programs use ^99m^Tc, with 30 million investigations performed each year.^[70]^ The ^99m^Tc radiotracers that are used for bone imaging do not require relatively close proximity to a medical cyclotron and a rapid distribution network, which usually costs about one-tenth of the cost of PET tracers. In addition, there is the potential for simultaneous multi-tracer studies with different radionuclides being used to examine different biologic pathways in a single imaging session. SPECT/CT systems also cost less than PET/CT systems and have a much greater installed base worldwide. For example, recent data from Europe revealed a 22% increase in the number of SPECT/CT scanners installed in France from 2015 to 2018, with similar growth figures in the UK and Germany.^[70]^

Standardization and quality control are key requirements for taking full advantage of the possibility of providing truly personalized and high-quality patient care, and many major professional associations and international institutions, such as the Society for Nuclear Medicine and Molecular Imaging, the European Association for Nuclear Medicine and the International Atomic Energy Agency, have proposed initiatives for developing robust and standard practice in SPECT/ CT imaging. This offers potential benefits for the study of gouty arthritis and it is likely that in the future SPECT/ CT imaging will be included in the evaluation criteria for diagnosing and staging this disease.

## Future and challenge

Gout is a treatable chronic disease, and the main strategy for effective management is to reverse the deposition of MSU crystals by uric acid reduction, and to prevent gout attacks, tophi deposition and complications, and thereby improve the quality of life.^[20]^

### Chronic kidney disease

Studies have shown that uric acid plays an independent role in the onset and development of chronic kidney disease (CKD).^[82]^ Gout and CKD often co-exist, and as many as about 70% of adults with gout have an estimated glomerular filtration rate (eGFR) of < 60 mL/ min/1.73m^2^, while 20%–24% have an eGFR of < 30 mL/ min/1.73m^2^.^[83]^ Decreased eGFR is a risk factor for early development of tophi, suggesting that renal function regulates the severity of gout. However, in clinical practice, there is a lack of information to guide the management of patients with CKD who have gout.^[83]^ This is partly due to the exclusion of patients with CKD from gout therapies, failure to report results stratified according to measures of renal function and inconsistency in the way measurements are obtained and reported,^[84]^ leading to conflicting recommendations from professional bodies regarding the treatment of patients with CKD and gout.^[85,86]^ Some clinical studies have provided evidence that urate lowering therapy (ULT) may help prevent and delay decreased renal function in patients with CKD.^[87]^ The gold standard for measuring renal function is to measure eGFR *via* inulin clearance, but the test is cumbersome, time-consuming, and cannot be routinely applied in the clinic. Accordingly, in clinical practice, eGFR is usually measured from serum creatinine levels, but this approach is not ideal.^[88]^ Studies have shown that ^99m^Tc-DTPA kidney SPECT/ CT is more reliable in measuring renal clearance and a promising way to measure eGFR.^[89]^ A topic that is currently of major interest in SPECT/CT research is the use of deep learning-based automated measurement of GFR.^[90]^ Moreover, kidney SPECT/CT can be used to assess both the filtration function of the glomeruli and the pathophysiology of the renal parenchyma,^[91]^ and we recommend that these assessments are added to the SPECT/CT imaging protocol for the management of patients with gout. In future, it will be possible to combine measurement of serum uric acid with kidney SPECT/ CT to predict the course of the disease in patients with CKD and gout.^[21]^

### Cardiovascular disease

There have been recent reports that elevated UA is associated with cardio-vascular (CV) disease and related to CV disease-related mortality,^[92]^ CV events (mainly acute coronary syndrome [ACS]) and stroke. In addition, UA is closely associated with heart failure^[93]^ and causes higher mortality,^[94]^ as well as the onset of atrial fibrillation.^[95]^ These clinically important findings have led to the measurement of UA being incorporated in the latest European guidelines for assessing arterial hypertension and stratification management for risk of CV.^[96]^

In recent years, it has been reported in one prospective study^[97]^ and one retrospective study^[98]^ that DECT can detect the deposition of MSU in blood vessels. Based on these studies, Khanna *et al*. suggested that MSU deposition on the wall of blood vessels may have a pro-inflammatory effect accelerating the onset of atherosclerosis, and increasing the incidence of CV events and strokes. Recent developments in dual-isotope SPECT imaging, utilizing ^99m^Tc-methoxy-isobutylisonitrile (MIBI) and the fatty-acid metabolism imaging agent ^123^I–beta-methyl-iodophenyl-pentadecanoic acid (BMIPP), have demonstrated that in patients with CV, functional metabolic changes can be observed in the distribution of myocardial injury caused by spasm of the distal vessels of the anterior descending branch of the left coronary artery.^[99]^ Since the pathological changes in metabolism occur before the changes in structure, SPECT/ CT may potentially be used to predict the deposition of MSU crystals in the coronary veins, so that proactively, interventions can be made to prevent the occurrence of coronary atherosclerosis.

In summary, with the development of contemporary precision medicine including the use of imaging techniques such as SPEC/CT, the individualized diagnosis and treatment of gout is likely to be significantly enhanced. In particular, application of new imaging hardware and deep learning algorithms and multi-disciplinary collaboration will all enhance the application of SPECT/CT imaging in diagnosing and monitoring treatment response in gouty arthritis. New developments that are in progress in physics/engineering, radiopharmaceuticals/chemistry, and design of new chelates and radiolabeled auxiliary groups will additionally allow more accurate visualization of the evolution of pathology and advances in the development of precision medicine for gouty arthritis.
